# Predictive Model for Outcomes in Inflammatory Bowel Disease Patients Receiving Maintenance Infliximab Therapy

**DOI:** 10.1093/crocol/otae052

**Published:** 2024-11-22

**Authors:** Rochelle Wong, Paris Charilaou, Amy Hemperly, Lihui Qin, Yushan Pan, Prerna Mathani, Randy Longman, Brigid S Boland, Parambir S Dulai, Ariela K Holmer, Dana Lukin, Siddharth Singh, Mark A Valasek, William J Sandborn, Ellen Scherl, Niels Vande Casteele, Robert Battat

**Affiliations:** Division of Gastroenterology and Liver Diseases, Department of Medicine, University of Southern California, Los Angeles, CA, USA; Department of Medicine, Section of Gastroenterology and Hepatology, Wake Forest Medical School, Charlotte, NC, USA; Division of Gastroenterology, Department of Medicine, University of California, San Diego, La Jolla, CA, USA; Department of Pathology, Weill Cornell Medicine, New York, NY, USA; Division of Gastroenterology and Hepatology, Department of Medicine, Weill Cornell Medicine, New York, NY, USA; Division of Gastroenterology and Hepatology, Department of Medicine, Weill Cornell Medicine, New York, NY, USA; Division of Gastroenterology and Hepatology, Department of Medicine, Weill Cornell Medicine, New York, NY, USA; Division of Gastroenterology, Department of Medicine, University of California, San Diego, La Jolla, CA, USA; Division of Gastroenterology and Hepatology, Department of Medicine, Northwestern University, Chicago, IL, USA; Division of Gastroenterology, Department of Medicine, NYU Langone Health, New York, NY, USA; Division of Gastroenterology and Hepatology, Department of Medicine, Weill Cornell Medicine, New York, NY, USA; Division of Gastroenterology, Department of Medicine, University of California, San Diego, La Jolla, CA, USA; Department of Pathology, University of California, San Diego, La Jolla, CA, USA; Division of Gastroenterology, Department of Medicine, University of California, San Diego, La Jolla, CA, USA; Division of Gastroenterology and Hepatology, Department of Medicine, Weill Cornell Medicine, New York, NY, USA; Division of Gastroenterology, Department of Medicine, University of California, San Diego, La Jolla, CA, USA; Department of Gastroenterology, University of Montreal, Montreal, Quebec, Canada

**Keywords:** endoscopy, pathology, predictive model, infliximab, maintenance

## Abstract

**Background:**

No models predict future outcomes in inflammatory bowel disease (IBD) patients receiving maintenance infliximab therapy. We created a predictive model for unfavorable outcomes.

**Methods:**

Adult patients with IBD receiving maintenance infliximab therapy at 2 centers with matched serum infliximab concentrations and blinded histologic scores (Robarts Histopathologic Index [RHI]) were included. The primary endpoint was an unfavorable outcome of active objective inflammation or need for IBD-related surgery or hospitalization at 6–18 months follow-up. Internal variables were identified using univariable analyses, modeling used multivariable analysis, and performance was assessed (area under receiver-operating curve [AUC]) and externally validated.

**Results:**

In 81 patients, 40.7% developed unfavorable outcomes at follow-up. Infliximab concentration <9.3 µg/mL (odds ratio [OR] 5.3, *P* = .001) and RHI > 12 (OR 3.4, *P* = .03) were the only factors associated with developing the primary unfavorable outcome. A prediction score assigning 1 point to each variable had good discrimination and performed similarly on internal (AUC 0.71) and external (AUC 0.73) cohorts. The risk of primary unfavorable outcomes in internal and external cohorts, respectively, was 23% and 15% for a score of 0, 46% and 50% for a score of 1, and 100% and 75% for a score of 2. Infliximab concentration alone performed similar to the 2-predictor model in internal (AUC 0.65, *P* = .5 vs. 2-predictor model) and external (AUC 0.70, *P* = .9, vs. 2-predictor model) cohorts.

**Conclusions:**

Using unbiased variable selection, a 2-predictor model using infliximab concentrations and histology identified IBD patients on maintenance infliximab therapy at high risk of future unfavorable outcomes. For practical applicability, infliximab concentrations alone performed similarly well.

## Graphical Abstract

**Graphical Abstract:**
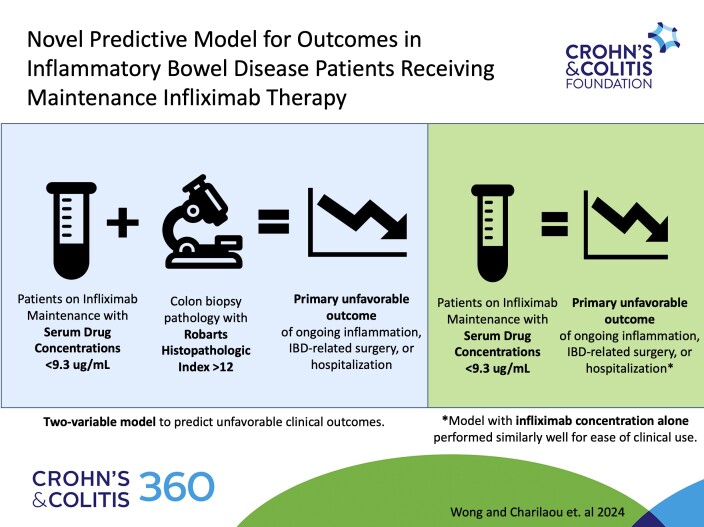

## Introduction

Infliximab is an effective induction and maintenance therapy for patients with Crohn’s disease (CD) and ulcerative colitis (UC).^1^ In many patients with inflammatory bowel disease (IBD), infliximab successfully induces clinical response. Clinical responders to infliximab are unique from those with primary nonresponse, and no predictive models exist once a patient has had sufficient improvement in clinical course to be able to proceed to standard maintenance therapy. Recent data have described persistent histologic inflammation to predict patient outcomes. However, multiple factors often interplay in determining outcomes, and data are lacking on a comprehensive model to predict patient outcomes in patients on infliximab maintenance therapy.

IBD disease activity endpoints include assessments of clinical symptoms, endoscopic inflammation, and histologic inflammation. Achieving both symptomatic and endoscopic remission is currently recommended.^2^ However, nearly one-third of those in clinical or endoscopic remission do not achieve histologic remission.^3,4^ Data suggest that histologic remission may better predict sustained clinical remission than endoscopic remission in patients with UC.^5^ Histologic healing has also been associated with lower rates of clinical relapse, corticosteroid use, hospitalizations, and IBD-related surgeries,^6,7^ while persistent histologic activity despite endoscopic remission has been associated with higher rates of relapse.^8^ Histologic indices, including the Robarts Histopathology Index (RHI), have been developed and validated to quantify histologic inflammation and can be used to identify treatment effects during drug maintenance therapy.^8,9^ Multiple studies have shown that RHI is a validated score in UC and CD.^10–12^ RHI is a responsive indicator of histologic disease and treatment response in CD,^13–15^ with similar test characteristics to other histologic indices^16^ and validated against endoscopy.^17,18^ Expert consensus statement agrees that RHI is appropriate for the assessment of histological disease in CD.^12^ Landmark clinical trials, such as the LOVE-CD trial, have also utilized the RHI for the assessment of histologic disease activity.^14,16,19^ Cross-sectional data have shown that higher tumor necrosis factor-antagonist trough concentrations are associated with clinical remission and mucosal healing.^20–24^ Previous studies have shown that pretreatment patient characteristics and peri-induction infliximab drug concentrations are associated with improved outcomes.^25^ However, no models exist using predictors of outcomes in the subgroup of patients with IBD who respond to induction therapy with infliximab and continue into maintenance therapy. In addition, no model has used multiple patient variables and included both validated histologic and drug concentration data to predict outcomes. Identifying patients at higher risk of clinical relapse may allow closer follow-up and better optimization of medical therapy through reactive or proactive therapeutic drug monitoring.^26,27^

Many patients with initial treatment response will develop unfavorable disease-related outcomes.^28,29^ This scenario after induction of infliximab therapy is frequently encountered by treating physicians. To guide care and address this major knowledge gap, the current study internally developed and externally validated a model to predict future outcomes for patients receiving maintenance therapy with infliximab. We assessed multiple variables, including serum drug concentrations and the RHI, a validated histologic disease activity index.

## Methods

### Patient Population

In this prospective study, adult patients (aged 18 or older) from 2 tertiary IBD centers treated between 2011 and 2020 with maintenance infliximab therapy (≥14 weeks after treatment initiation) with serum trough concentrations within 90 days of colonoscopy and biopsy were included. Data were obtained at any point during maintenance therapy, and then the patient was followed longitudinally from the baseline collection date. Data were extracted from patient charts for an internal training cohort and an external validation cohort. Those with ileostomy or ileal pouch-anal anastomosis were excluded.

Demographics such as age, gender, disease type, comorbidities, and medication history, including baseline steroid and immunomodulator use, dose, frequency, and duration of infliximab therapy, were collected. Disease characteristics, including Montreal disease classification, baseline active endoscopic disease score, baseline fecal calprotectin (FCP), baseline C-reactive protein (CRP), and presence of antidrug antibodies, were also collected. As is standard in clinical trials and practice, biopsies were taken from endoscopically inflamed segments for both CD and UC. In the presence of inflammation or ulcerations, biopsies were taken from the edge of the ulcer. It is also standard and recommended in both trials and translational research that biopsies of noninflamed tissue are then collected at random. In our cohort, if no endoscopic inflammation was observed, random segmental biopsies were taken from each segment throughout the ileum and/or colon. For UC patients, at least 1 biopsy was obtained from the rectum, given the continuous pattern of inflammation starting from the rectum in this disease.

Baseline clearance was utilized as a variable and calculated using the following: UC baseline infliximab clearance (L/d)^30^ = 0.407 × (albumin/4.1)^−1.54^ × (1.471)^ATI^ × (0.764)^sex^, where albumin is albumin concentration (g/dL) at baseline, ATI is antibodies to infliximab, 1 = yes, 0 = no, and sex = 1 for females, 0 for males. CD clearance^31^ was calculated = 5.42 × (WGT/65)^−0.313^ × (ALB/4.1)^−0.855^ × (1.292)^ATI^ × (0.863)^IMM^, where WGT is the weight (kg), ALB is albumin concentration at baseline (g/dL), and IMM = 1 in patients receiving immunomodulators and IMM = 0 in patients not receiving immunomodulators. Clinical outcomes data such as IBD-related hospitalization, IBD-related surgery, and follow-up endoscopic disease activity score, fecal calprotectin, and/or CRP were collected at 6–18 months after the baseline date of serum drug concentration measurement.

### Robarts Histopathologic Index

For baseline histological data, a blinded pathologist used the RHI to provide histologic scoring of biopsies obtained from the colonoscopy within 90 days of baseline trough concentration. The RHI was first developed by Mosli et al. in 2017, as a derivation from the Geboes score.^9^ It scores four histologic features on a grade of 0–3 on the same scale as the Geboes score, with different weights for each feature: Lamina propria chronic inflammation, lamina propria neutrophils, epithelial neutrophils, and surface epithelial injury or erosion or ulceration. The total score can range from 0 (no disease activity) to 33 (most severe disease activity). Chronic inflammation is the lowest weighted feature, while erosion or ulceration is the highest weighted. The formula for calculating Robarts’ Histopathology Index is as follows: RHI = (1 × chronic inflammatory cell infiltrate grade) + (2 × lamina propria neutrophils grade) + (3 × epithelial neutrophils grade) + (5 × erosion or ulceration grade).

### Serum Trough Drug Concentration

For patients with multiple serum trough (≤6 days) drug concentrations in the proximity of endoscopic assessment, a single sample with the closest date to endoscopy was included. Trough concentrations were a priori defined as 6 days or less prior to infusion. Patients who discontinued infliximab (*n* = 16) after drug-level measurement were included only if they had a clinical outcome >90 days after baseline, were off infliximab <90 days, and were not receiving any new therapy for >90 days. Serum drug concentrations were measured by homogenous mobility shift assay (HMSA, Prometheus Laboratories Inc.). Patients were monitored using standard therapeutic drug monitoring while on maintenance therapy during the follow-up period, and doses were adjusted based on those levels to achieve therapeutic range.

### Endpoints

The primary endpoint (unfavorable outcome) was the need for IBD-related surgery, hospitalization, or signs of objective inflammation 6–18 months after baseline serum infliximab trough concentration. The presence of objective inflammation was defined as endoscopic activity simple endoscopic score (SES-CD) > 6 for CD patients^32^ or Mayo endoscopic score (MES) ≥ 2 for UC patients. In patients without formal endoscopic scoring, patients were categorized to be in endoscopic remission when an impression of complete normalization or remission was documented by the endoscopist. If this endoscopic data were unavailable, fecal calprotectin (FCP) ≥ 150 µg/g was utilized. CRP above the upper limit of normal (≥1.0 mg/dL) for training cohort institutions was used if FCP was unavailable. Patients whose fecal calprotectin or CRP was elevated in the setting of acute infection were excluded. Secondary outcomes of clinical (symptomatic) remission and endoscopic remission were also evaluated. Symptomatic remission was evaluated by the Harvey–Bradshaw Index for CD and the modified Ulcerative Colitis Disease Activity Index for UC.

### Statistical Analysis and Model Development

Descriptive analysis was done using chi-square and *t*-test/Mann–Whitney tests for categorical and continuous predictors, respectively. For normally distributed variables, mean and standard deviation (SD) were reported. For non-normally distributed variables, medians and interquartile range (IQR) were reported.

Univariable analysis with logistic regression for screening predictors of the primary outcome was initially performed, and predictors with *P* < .10 (defined a priori) were subsequently included in the multivariable regression model.^33,34^ The prediction model was developed based on published guidelines.^35^ Cutoffs for infliximab drug concentrations and RHI scores were determined using receiver-operator-characteristic area under the curve (AUROC), and the threshold used was selected based on the balance between sensitivity and specificity for the primary outcome. To avoid multicollinearity, variance inflation factors (VIF) were evaluated (goal VIF < 10 for each predictor). The Hosmer–Lemeshow test was used to assess for model calibration. From the multivariable model, points were assigned for individual variables (described later), AUROC analysis using the scoring system was used to assess the model’s discriminatory performance, and a cross-validated AUC was calculated using the internal cohort to assess for model overfitting.

The prediction model was trained on the internal cohort, which was subsequently cross-validated (4 folds) and externally validated on a different institution patient cohort. The model was converted into a clinical prediction score by assigning equal points per each predictor’s odds ratios (ORs) and dividing them by their greatest common factor. The reported AUCs were based on the score-based evaluation. The observed and predicted probability of the primary outcome in both internal and external cohorts was compared among each possible score. Secondary analyses included evaluating the model performance for individual components of the composite primary outcome (ie, objective remission, hospitalization, surgery). As insufficient outcome events existed individually for surgery and hospitalization, model performance was evaluated for objective remission alone. This was performed in a joint (both internal and external) cohort to assess performance with adequate outcome events. Additionally, we explored the performance of using drug concentration alone as compared to utilizing the complete model to predict patient outcomes.

## Results

### Patient Demographics and Endpoint Prevalence

A total of 81 patients (internal training cohort: *n* = 48, external validation cohort: *n* = 33) were included (Table 1). All patients had both baseline infliximab drug concentrations, baseline endoscopic evaluation, and baseline RHI score. Forty CD patients, 38 UC patients, and 3 indeterminate colitis were included. The mean (±SD) age was 38.0 years (±15.9), mean time of trough concentration measurement prior to infusion was 3.5 days (±7.8), with all >4 weeks from prior infusion. The mean time from treatment initiation was 116.8 weeks (±133.1). No patients were on immunomodulator therapy at the time of drug concentration measurement. No patients had an acute disease exacerbation at the time of serum drug concentration. No patients had primary nonresponse. The median follow-up time from baseline drug level to the primary outcome was 328 days (251–426) and was similar between cohorts. Infusions were administered on average 1.21 (±0.58) mg/kg/week. The median (IQR) infliximab drug concentration was 12.4 (6.9–29) µg/mL. Thirty-one patients (38.3%) total out of 81 had baseline endoscopic remission at the time of drug concentration, defined as SES-CD < 3, MES = 0, and no ulcers. The median RHI score was 7 (0–16). Nine patients out of 79 (11.4%) with available data had antibodies to infliximab. Fifty-nine patients (72.8%) had no previous use of biologic therapy, while 17.3% (14/81) had at least 1 previous biologic use. Eighteen patients (22.2%) had previous IBD-related surgery. A total of 40.7% (33/81) patients developed the unfavorable primary composite outcome at follow-up, 32.5% of patients developed objective signs of inflammation, 9.9% required IBD-related hospitalization, and 7.4% required IBD-related surgery. Five patients had both hospitalization and surgery. Of patients with endoscopic evaluations, none had SES-CD scores between 3 and 6 or MES of 1. Of patients with fecal calprotectin evaluations, none had a value between 50 and 150 µg/g.

**Table 1.** Cohort description.

	Internal (*n* = 48)	External (*n* = 33)	Joint (*n* = 81)
Demographic characteristics			
Age, years			
Mean (SD)	38.0 (15.9)	31.9 (22.9)	38.0 (15.9)
Median (range)	33 (26–48)	24 (16–37)	33 (26–48)
Female sex, *n* (%)	26 (54.2%)	14 (42%)	40 (49.4%)
Types of IBD			
CD, *n* (%)	21 (43.8%)	19 (57.6%)	40 (49.4%)
L1 ileal	2 (10.0%)^a^	3 (15.8%)^b^	5 (13.2%)^c^
L2 colonic	3 (15%)^a^	7 (36.8%)^b^	10 (26.3%)^c^
L3 ileocolonic	13 (65%)^a^	8 (44.4%)^b^	21 (55.3%)^c^
L4 isolated upper GI disease	2 (10%)^a^	0 (0%)^b^	2 (5.3%)^c^
B1 nonstricturing, nonpenetrating	11 (57.9%)^d^	8 (44.4%)^b^	19 (51.4%)^e^
B2 stricturing	1 (5.3%)^d^	4 (22.2%)^b^	5 (13.5%)^e^
B3 penetrating	7 (36.8%)^d^	5 (27.8%)^b^	12 (32.4%)^e^
UC, *n* (%)	24 (50.0%)	14 (42.4%)	38 (46.9%)
Proctitis (rectum/anus only)	4 (16.7%)	2 (14.3%)	6 (15.8%)
Left-sided (rectum to splenic flexure)	13 (54.2%)	3 (21.4%)	16 (42.1%)
Extensive (beyond splenic flexure)	7 (29.2%)	9 (64.3%)	16 (42.1%)
Indeterminate	3 (6.3%)	0 (0%)	3 (3.7%)
Baseline data			
Baseline CRP > 1	12 (26.7%)	3 (11.5%)	15 (21.1%)
Baseline FCP > 50	8 (36.7%)	Data not available	Data not available
Baseline median SES-CD	2 (IQR: 0–15)^f^	1 (IQR: 0–2.5)^h^	1 (IQR: 0–4)^i^
Baseline median MES	2 (IQR: 0.75–2.25)^g^	1 (IQR 0.5–2.5)^f^	2 (IQR: 0.5–2.5)^j^
Baseline any endoscopic remission (SES-CD < 3, MES 0, no ulcers)	14 (29.2%)	17 (51.5%)	31 (38.3%)
No previous biologic	38 (79.2%)	21 (63.6%)	59 (72.8%)
At least 1 previous biologic	10 (20.8%)	12 (36.4%)	22 (27.2%)
Any previous anti-TNF	5 (10.4%)	12 (36.4%)	17 (21.0%)
Previous IBD surgery	8 (16.7%)	10 (30.3%)	18 (22.2%)
Infliximab therapy			
Median trough drug concentration (µg/mL) (IQR)	12.1 (3.3–22.1)	13.3 (8.5–30.6)	12.4 (6.9–28)
Mean mg/kg/week	1.26 (0.58)	1.14 (0.57)	1.21 (0.58)
Mean therapy duration (weeks)	109.8 (105.2)	127.0 (166.3)	116.9 (133.1)
Mean timing of drug level prior to next infusion (days)	0.39 (1.5)	7.7 (10.6)	3.5 (7.8)
Histologic scoring			
RHI score (median, IQR)	6 (0–11.25)	7 (0–17)	7 (0–16)
Outcomes 6–18 months			
Unfavorable primary composite outcome (*n*, %)	20 (41.7%)	13 (39.4%)	33 (40.7%)
Presence of objective inflammation (*n*, %)	15 (31.9%)	11 (33.3%)	26 (32.5%)
IBD-related hospitalization (*n*, %)	5 (10.4%)	3 (9.1%)	8 (9.9%)
IBD-related surgery (*n*, %)	4 (8.3%)	2 (6.1%)	6 (7.4%)

Abbreviations: CD, Crohn’s disease; CRP, C-reactive protein; FCP, fecal calprotectin; IBD, inflammatory bowel disease; IQR, interquartile range; MES, Mayo Endoscopic Score; RHI, Robarts Histopathologic Index; SD, standard deviation; SES-CD, simple endoscopic score for CD patients; TNF, tumor necrosis factor; UC, ulcerative colitis.

^a^Total 20 patients with available data.

^b^Total 18 patients with available data.

^c^Total 38 patients with available data.

^d^Total 19 patients with available data.

^e^Total 37 patients with available data.

^f^Total 11 patients with available SES-CD or MES score data.

^g^Total 12 patients with available MES score data.

^h^Total 15 patients with available SES-CD score data.

^i^Total 26 patients with available SES-CD score data.

^j^Total 23 patients with available MES score data. All patients had endoscopic impression data available to derive remission status.

### Univariable Analyses for the Primary Outcome

Univariable analyses for the primary outcome are outlined in Table 2. An infliximab drug concentration of <9.3 µg/mL and RHI > 12 were found to be the optimal cutoff for the primary outcome using AUROC analyses (Supplementary Figure 1). On the internal cohort, infliximab drug concentration <9.3 µg/mL and RHI score >12 were the only predictors of the primary outcome on univariable analysis with a *P* < .1 for inclusion in the multivariable analyses. Importantly, neither disease type (CD vs. UC: OR = 0.9; 95% CI, 0.3–2.9; *P* = 0.9), baseline endoscopic activity, predicted drug clearance, nor C-reactive protein was associated with the development of the primary outcome. In addition, proportions with the primary outcome were numerically similar between those with (40%) and without (39%) baseline clinical remission status, with (33%) and without (42%) immunomodulators, and with (36%) and without (41%) corticosteroids.

**Table 2.** Univariable analysis for primary unfavorable outcome on internal training cohort.

Predictor	OR (95% CI)	*P*-value
IFX drug level < 9.3 μg/mL	**3.6 (1.1–12.3)**	**.04**
Crohn’s disease	0.9 (0.3–2.9)	.85
RHI score > 12	**3.7 (0.9–14.9)**	**.07**
Baseline active endoscopic disease	3.3 (0.8–13.8)	.11
Baseline infliximab clearance (mL/day)	0.13 (0–35.0)	.47
C-reactive protein (mg/dL)	0.4 (0.1–1.4)	.14
Any prior biologic use	0.31 (0.06–1.7)	.17

Abbreviations: CI, confidence interval; CRP, C-reactive protein; CV, cross-validated; IBD, inflammatory bowel disease; IFX, infliximab; IMM, immunomodulator; OR, odds ratio; RHI, Robarts Histopathological Index; ROC-AUC, receiver-operating characteristic area under the curve.

IFX drug level and RHI were determined using ROC-AUC at univariate analysis at the maximal point of harmonic mean of F1 score and accuracy.

### Prediction Model Derivation, Evaluation, and Validation

**Figure 1.**
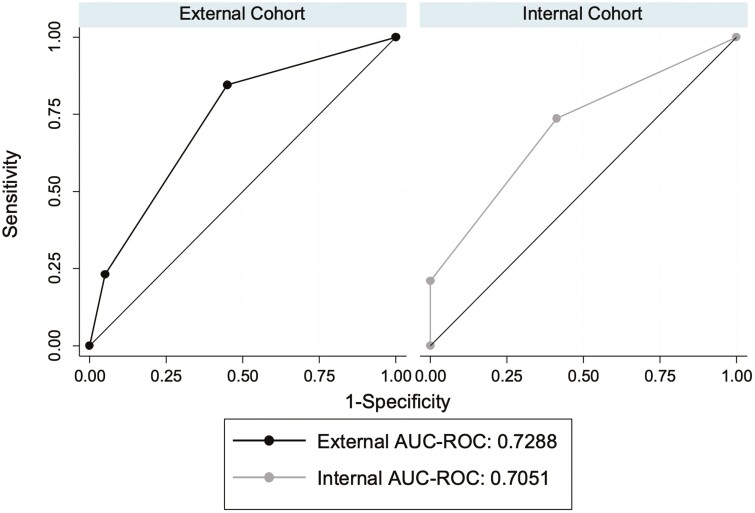
Prediction model had good discrimination for primary outcome on both the internal and external cohort.

The 2 covariates with *P* < .1 from univariable analyses in the training (internal) cohort were used in multivariable analyses to predict the primary outcome (Table 3). On multivariable analysis, infliximab drug concentration <9.3 µg/mL was significantly associated with the development of the primary outcome (OR 4.3; 95% CI, 1.2–16.0; *P* = .03) and RHI score >12 (OR 4.6; 95% CI, 0.91–21.0; *P* = .05) numerically favored the development of the primary outcome in the training (internal) cohort. Using these 2 variables weighted for the model (infliximab drug concentration <9.3 µg/mL: 1 point, RHI score >12: 1 point), the clinical prediction score on the internal cohort had good discrimination for the primary outcome (AUC 0.71; 95% CI, 0.57–0.84, Figure 1) and was well-calibrated (Hosmer–Lemeshow test, *P* = .505). The cross-validated AUC was 0.73 (95% bootstrap bias-corrected CI, 0.52–0.89), suggesting no significant overfitting (Δ-AUC *P* = .83). In the joint cohort (*n* = 81), infliximab drug concentration <9.3 µg/mL (OR 5.3; 95% CI, 1.9–14.8; *P* = .001) and RHI score >12 (OR 3.4; 95% CI, 1.1–10.3, *P* = .03) were both significantly associated with the development of the primary outcome.

**Table 3.** Multivariable analysis for primary unfavorable outcome on internal training cohort.

Predictor	OR (95% CI)	*P*-value
IFX drug level < 9.3 µg/mL	4.3 (1.2–16.0)	.03
RHI score > 12	4.6 (0.91–21.0)	.05

Abbreviations: CI, confidence interval; CV, cross-validated; IBD, inflammatory bowel disease; IFX, infliximab; OR, odds ratio; RHI, Robarts Histopathological Index; ROC-AUC, receiver-operating characteristic area under the curve.

Scoring system (OR rounded to closest 0.5 and divided by 4.5): each variable = 1 point. Main model: AUROC 0.70 (95% CI, 0.55–0.85); CV-AUROC = 0.71 (bootstrap bias-corrected 95% CI, 0.51–0.88). Scoring model: AUROC 0.71 (95% CI, 0.57–0.84); CV-AUROC = 0.73 (bootstrap bias-corrected 95% CI, 0.52–0.89). Hosmer–Lemeshow test for calibration *P* = .505 (passed calibration), mean variance inflation factor 1.08.

In the external validation cohort, the predictive scoring model also demonstrated good discrimination for the primary outcome (AUC 0.73; 95% CI, 0.52–0.89; Figure 1). The performance characteristics of the model on the external cohort were as follows: sensitivity 85%, specificity 55%, positive-predictive value (PPV) 55%, negative-predictive value (NPV) 85%, accuracy 67%, and diagnostic OR 6.7.

**Figure 2.**
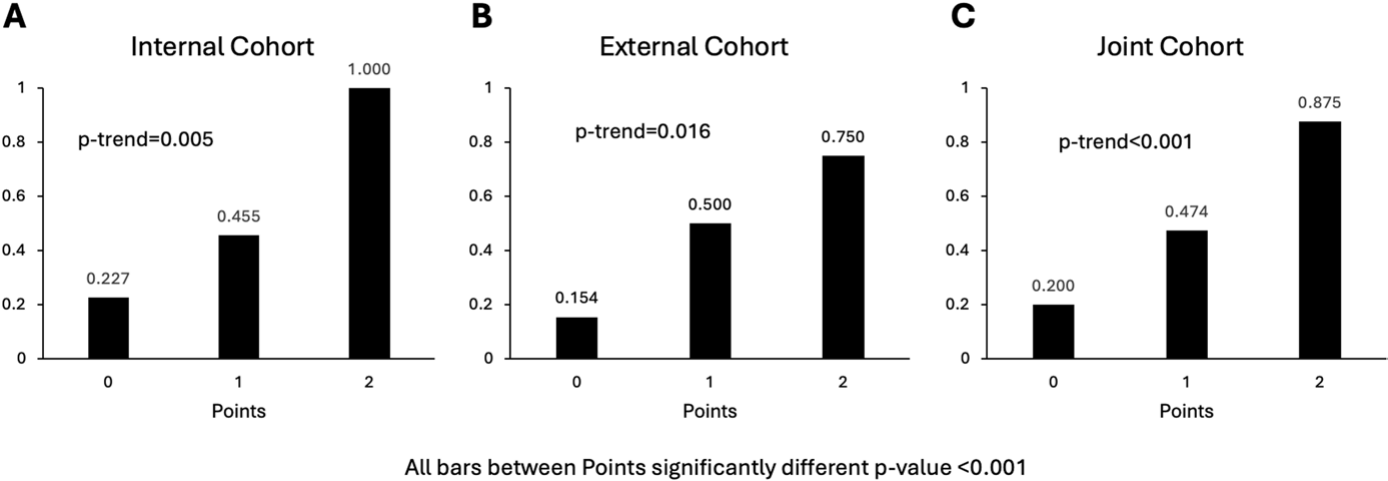
Observed composite outcome risk by prediction model points for (A) internal, (B) external, and (C) joint cohorts.

An observed score of 0 predicted risk of 23% and 15%, a score of 1 risk of 46% and 50%, and a score of 2 risk of 100% and 75% for the primary outcome in the internal and external cohorts, respectively. Risk increased significantly with increasing score (*P*trend = .016, Figure 2). Primary outcome risk was similar between each of the 3 scores when comparing the external cohort with the internal cohort. The observed and predicted risks for the primary outcome in both the internal and external cohorts were similar across each score (0, 1, and 2), confirming the model’s calibration (Supplementary Figure 2).

### Evaluation of Model Performance on Objective and Symptomatic Remission

Evaluation of the predictors on objective inflammation alone passed calibration and demonstrated good discrimination (AUC 0.71; 95% CI, 0.60–0.82; infliximab drug concentration <9.3 µg/mL: OR 3.7, 95% CI, 1.3–10.8, *P* = .02, RHI > 12: OR 4.4, 95% CI, 1.5–13.4, *P* = .008), PPV 75%, NPV 74%, accuracy 74%, with a diagnostic OR 8.4.

However, evaluation of the predictors on lack of symptomatic remission alone did not show any statistical significance (model discrimination AUC 0.54; 95% CI, 0.36–0.71).

### Performance of Infliximab Drug Concentration Alone

Among the internal (AUC 0.65; 95% CI, 0.51–0.79) and external (AUC 0.70; 95% CI, 0.52–0.88) cohorts, infliximab drug concentration alone demonstrated good discrimination, which was similar to the final 2-predictor model (comparison of δAUC: internal cohort *P* = .5, external cohort: *P* = .9, Supplementary Figure 3). Infliximab drug concentration alone had 62% sensitivity and 80% specificity for the primary outcome of the testing (external) cohort.

## Discussion

There is a lack of tools for physicians to risk stratify patients receiving maintenance therapy with infliximab. As opposed to patients who are predicted to have unfavorable outcomes prior to treatment initiation, patients who initially respond to infliximab and receive maintenance therapy are a unique group for which few predictive data exist. To address this need, the current study developed and validated a model to predict outcomes in these patients. Analyses of multiple variables revealed 2 predictors meeting the criteria for model inclusion. Uniquely, the model was accurate for important objective outcomes and used trough infliximab concentrations matched to the validated histologic scoring index (RHI). This model was internally trained and externally validated with good discrimination. The current model was able to differentiate the probability of an unfavorable outcome from 15% to 23% with a score of 0 to 75%–100% with a score of 2 in both cohorts. The model was additionally tested and performed similarly well for future intestinal inflammation quantified by objective measures. Interestingly, the performance of maintenance infliximab concentrations alone was explored for ease of practical application and performed similar to the 2-variable model. Importantly, outcomes were nearly identical between patients with CD and UC. Patients identified at higher risk for unfavorable outcomes during maintenance therapy may warrant closer follow-up or endo-histologic surveillance to titrate their therapy to target and prevent the development of poor outcomes. Earlier detection of loss of response to maintenance IFX therapy may also prompt escalation or change in biological therapy sooner than the current standard of care. As evidenced in the PROFILE trial, earlier aggressive care results in better patient outcomes.^36^ Thus, tools such as this model will be important to identify.

Endo-histologic mucosal healing, defined using both endoscopy and histology, is an emerging therapeutic endpoint in IBD.^37^ However, discordance can exist between endoscopic and histologic findings.^6,38^ The Selecting Therapeutic Targets in Inflammatory Bowel Disease (STRIDE) II guidelines maintain symptomatic and endoscopic remission as therapeutic targets, although histological healing is outlined as an important adjunctive treatment measure.^37^ However, data are lacking on the use of histologic inflammation as a predictive measure of outcomes in those subsequent to induction therapy. While the ideal definition of histologic remission is also still unclear,^37^ histologic disease activity indices such as the RHI^9^ and Nancy Histological Score^39^ are optimal indices validated in the literature in order to measure histologic inflammation.

The relationship between histologic remission and serum infliximab drug concentrations has previously been explored cross-sectionally at a single time point. However, these previous studies neither used validated histologic scoring nor utilized these data to predict future outcomes. Importantly, these studies found maintenance infliximab concentrations (≥9.8 μg/mL) that were similar to the current study (9.3 µg/mL) to be associated with histologic remission.^40–42^

Identifying patients at higher risk of clinical relapse may allow closer follow-up and better optimization of medical therapy. The model in the current study raised the probability of an unfavorable outcome occurring 60%–77%, comparing the highest and lowest scores. The prognostic value of histology has been studied in both UC and CD. Azad et al. used the Geboes score to predict 57.5% clinical relapse in a 12-month follow-up period of patients with quiescent UC based on increased numbers of eosinophils and lamina propia neutrophils.^43^ Bessissow et al. subsequently found that in patients with complete mucosal healing, a Geboes score of ≥3.1 predicted a higher risk of clinical relapse (20%).^44^ However, histologic inflammation of the intestine is related to multiple factors, including patient characteristics and medications. The current study analyzed these factors in addition to histology. These included demographics, drug clearance-related factors, pharmacokinetic factors (ie, serum drug concentrations), and various baseline disease activity measures (ie, endoscopy, biomarkers). While previous studies lacked standardization in histologic score reporting or definitions of remission,^45^ the current study used the RHI score, a validated histologic index, to develop an outcomes predictor model while a patient is on maintenance infliximab therapy.^46^

Strengths of the current study include using trough infliximab concentrations matched to validated endoscopic and histologic disease activity indices. We defined complete endoscopic remission stringently. All patients with objective inflammation had an SES-CD > 6 for Crohn’s patients and MES of 2 or more for UC patients or FCAL > 150. As no patients had neither an SES-CD between 3 and 6, MES of 1, nor FCAL 50–150 µg/g, all patients with absence of objective inflammation had SES-CD < 3 or MES = 0 or FCAL < 50 µg/g. In addition, patients had data collected longitudinally in 2 separate cohorts for internal training and external validation.

Limitations of the study include the relatively small patient number utilized to train and externally validate the model. The small sample size does limit our evaluation of predictors of unfavorable primary outcomes, as other predictive factors of adverse outcomes may exist and could potentially be identified in a sufficiently powered analysis using a larger patient sample. However, we utilized 2 separate patient cohorts, the second of which to externally validate the model, and performed extensive detailed analysis to identify our two significant predictors (infliximab drug concentration and RHI). Another limitation is missing endoscopic data and data derivation from tertiary centers, potentially not reflecting community practice. However, for those with missing endoscopic data, a robust and widely accepted objective marker of intestinal inflammation (fecal calprotectin) was utilized.^47^ In addition, important outcomes of hospitalization and surgery were analyzed. Of note, there were no observations with infliximab concentrations between 9.3 and 11.3 µg/mL, and thus a possibility exists that a conservative estimate for a concentration <11.3 µg/mL may portend to negative outcomes. Similarly, RHI values between 12 and 18 were lacking, and an RHI > 18 may be the threshold utilized as well. Sample size may have affected conclusions on the internal cohort for the impact of RHI on outcomes (*P* = .05), which was statistically significant when analyzed in the larger joint cohort (*P* = .03). A small minority of patients were misclassified using the model. However, the consistency of the data between cohorts added to the validity of the model: Event rates were similar for each score and in each cohort, and the event rate increased similarly as the score increased. The internal cohort increased from 23% with a score of 0%–100% for a score of 2, and the external cohort increased from 15% with a score of 0%–75% for a score of 2.

No predictive data exist for patients who have a sufficient clinical course to enter the maintenance phase of therapy. This study internally developed and externally validated a predictive model to identify the development of negative outcomes of objective inflammation, hospitalization, and surgery in patients on infliximab maintenance therapy. Two variables met the criteria for model inclusion. This model uniquely uses maintenance infliximab concentrations matched to a validated histologic disease activity index in addition to robust evaluation of multiple potential variables to predict outcomes.

## Conclusions

This study provides useful and clinically relevant information for practitioners to predict unfavorable outcomes in patients on infliximab maintenance therapy, using infliximab drug concentration and the validated RHI. In particular, the use of infliximab concentration alone (without histology) can also be an indicator of future unfavorable outcomes.

## Supplementary Material

otae052_suppl_Supplementary_Figures

## Data Availability

The datasets used and analyzed in the current study are available from the corresponding author upon reasonable request.
