# Supplementary material for: Predictive Model for Outcomes in Inflammatory Bowel Disease Patients Receiving Maintenance Infliximab Therapy
Source: Crohns Colitis 360. 2024 Nov 22;6(4):otae052. doi: 10.1093/crocol/otae052 (PMC11645457; doi:10.1093/crocol/otae052)
Supplement: otae052_suppl_Supplementary_Figures [file otae052_suppl_supplementary_figures.docx]

Supplementary Figure 1: **ROC-AUC Cutoff Values for IFX drug level and RHI.**


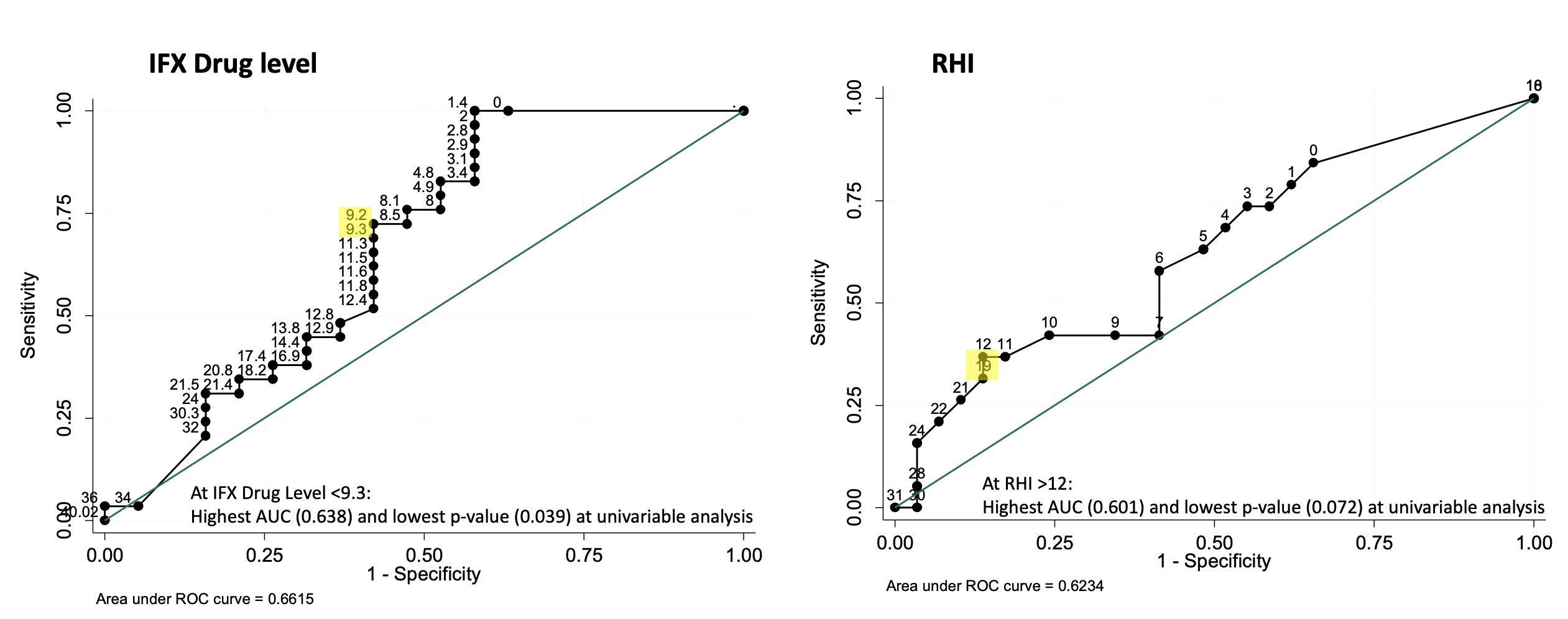


Supplementary Figure 2: **Model showed good calibration between observed and predicted risks for the primary outcome in both the internal and external cohorts.**


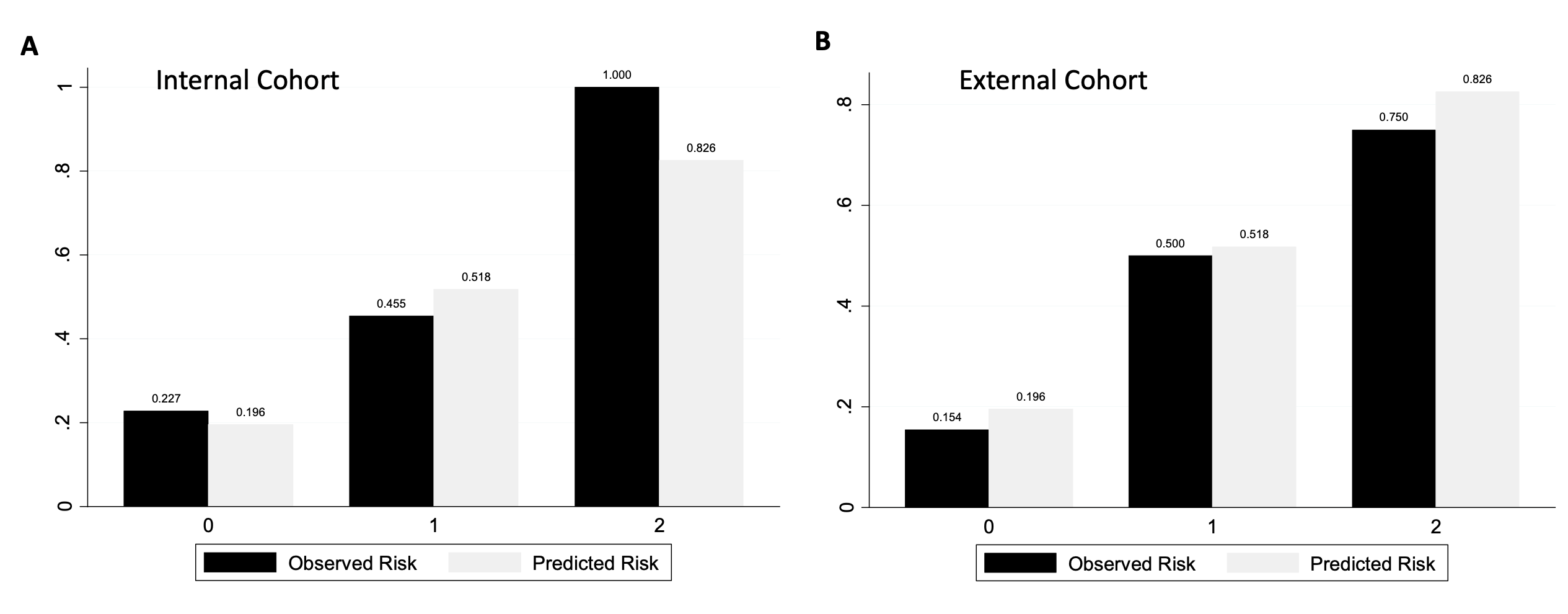


Supplementary Figure 3: **Prediction model using IFX Drug Level alone demonstrated similarly good discrimination compared to the final 2-predictor model.**
